# A randomized trial of video self-instruction in cardiopulmonary resuscitation for lay persons

**DOI:** 10.1186/1757-7241-21-36

**Published:** 2013-05-10

**Authors:** Rachel Godfred, Ella Huszti, Deborah Fly, Graham Nichol

**Affiliations:** 1 University of Washington-Harborview Center for Prehospital Emergency Care, Seattle, WA, USA

**Keywords:** Public, Cardiopulmonary resuscitation, Cardiac arrest, Education, Randomized trial

## Abstract

**Background:**

Cardiopulmonary resuscitation (CPR) improves outcomes after cardiac arrest. Much of the lay public is untrained in CPR skills. We evaluated the effectiveness of a compression-only CPR video self-instruction (VSI) with a personal manikin in the lay public.

**Methods:**

Adults without prior CPR training in the past year or responsibility to provide medical care were randomized into one of three groups: 1) Untrained before testing, 2) 10-minute VSI in compressions-only CPR (CPR Anytime, American Heart Association, Dallas, TX), or 3) 22-minute VSI in compressions and ventilations (CPR Anytime). CPR proficiency was assessed using a sensored manikin. The primary outcome was composite skill competence of 90% during five minutes of skill demonstration. Evaluated were alternative cut-points for skill competence and individual components of CPR. 488 subjects (143 in untrained group, 202 in compressions-only group and 143 in compressions and ventilation group) were required to detect 21% competency with compressions-only versus 7% with untrained and 34% with compressions and ventilations.

**Results:**

Analyzable data were available for the untrained group (n = 135), compressions-only group (n = 185) and the compressions and ventilation group (n = 119). Four (3%) achieved competency in the untrained group (p-value = 0.57 versus compressions-only), nine (4.9%) in the compressions-only group, and 12 (10.1%) in the compressions and ventilations group (p-value 0.13 vs. compressions-only). The compressions-only group had a greater proportion of correct compressions (p-value = 0.028) and compressions with correct hand placement (p-value = 0.0004) compared to the untrained group.

**Conclusions:**

VSI in compressions-only CPR did not achieve greater overall competency but did achieve some CPR skills better than without training.

## Background

Out-of-hospital cardiac arrest (OHCA) is common and lethal [1]. Although external chest compressions coupled with ventilations (CPR) may be lifesaving [2], many members of the general public are not well trained in these techniques, have trouble retaining the skills, or are reluctant to perform them [3]. Bystander CPR is frequently not performed [4]. Studies in animal models of arrhythmic arrest suggest that compressions-only CPR may be as effective as standard CPR [5]. Video self-instruction in compressions and ventilations training including watching a video while practising with a personal manikin achieved initial competency equivalent to standard CPR and retention performance at least as good as with traditional instructor-led training [6-8]. Broad implementation of such programs could improve the likelihood of bystander CPR as well as the likelihood of survival after OHCA. But the efficacy of video self-instruction in compressions-only CPR in lay people has not been rigorously compared to video self-instruction in compressions and ventilations or to no training. Therefore, we sought to compare skills of the lay public with immediate testing (i.e. untrained), or ten-minute video self-instruction in compression-only CPR with a personal manikin (CPR Anytime, American Heart Association, Dallas, TX), or 22-minute video self-instruction in compressions and ventilations with a personal manikin (CPR Anytime).

## Methods

This study was conducted in Seattle, Washington. The study protocol was approved by the University of Washington Institutional Review Board. Consent was obtained from all subjects.

### Subjects and recruitment

Volunteers were recruited by advertisement in the community. Subjects did not receive any benefit except the knowledge of life-saving CPR skills. Subjects completed and returned consent forms in person. Included were laypersons aged at least 18 years who were willing to be trained to respond to episodes of OHCA. Excluded were individuals who had participated in CPR training in the last year, and those with a responsibility to provide medical assistance in emergencies as their primary job description. The latter were defined as law enforcement officers, first responders, firefighters, paramedics, nurses, or physicians. Baseline demographic information was recorded, including age, gender, race, ethnicity, and educational attainment, and whether any first-degree relatives had heart disease. Enrollment was conducted between June, 2008 and August, 2010.

### Randomization

The study employed an experimental design with a control group untrained before testing (untrained), video self-instruction compressions-only group (compressions-only), and a compressions and ventilation video self-instruction group (compressions and ventilations). After providing informed consent, subjects were assigned randomly to one of the three groups, with an allocation ratio 1:√2:1 for increased efficiency in comparing two groups (untrained as well as compressions and ventilations) to a common group (compressions-only) [9].

### Intervention

Individuals were enrolled in the study one at a time. Two different rooms were used in this study: a waiting/reception room, and a training/testing room. Several measures were taken to minimize subjects’ exposure to information about the study. Subjects started off seated in a waiting/reception area that was isolated from the training/testing room to mask any incidental transfer of sound. Study materials and rooms were concealed from view when not in use. Upon entering the training/testing room, subjects were given the learning kit they were allocated to receive, if any, and were encouraged to watch the video and practice skills before the skills testing. After sufficient time to practice while watching, subjects allocated to the compressions-only or compressions and ventilation groups demonstrated their CPR skills for five minutes using a sensored manikin (Resusci-Anne with Skill Reporter, Laerdal Medical Inc., Wappinger Falls, New York). Subjects allocated to the untrained group underwent immediate demonstration of CPR skills. At the time of skill testing, subjects were told to imagine that the manikin was a person, whom they had just found on the ground unresponsive and not breathing, and to show what they would do. All subjects received a video-self instruction kit for their ongoing personal use upon completion of the skills testing.

### Performance assessment

CPR skills were assessed based on adequacy of rate and depth of compressions, and hand placement during compressions with a sensored manikin. Adequate number was defined as an average of between 80 to 100 compressions per minute over five minutes; adequate depth was defined as an average depth of 38 to 52 mm over five minutes; [10] adequate hand position was defined as midline between the nipples. Each of these components were categorized as adequately or inadequately performed.

### Outcomes

The binary primary outcome of CPR competency was defined at a subject level as 90% or greater ‘correct’ compressions. A ‘correct’ compression was defined as having all three components (rate, depth and hand placement) adequately performed. Secondary outcomes included adequacy of each of the three individual components of CPR, defined as: compression rate (number/minute); compression depth (in millimeters) and proportion of compressions with depth ≥38 mm; as well as the proportion of compressions with correct hand placement.

### Sample size

In a previous trial of video self-instruction in CPR, instructors assessed CPR competence as 7% in the untrained group [8]. Assuming 7% competency without training, α = 0.05, power = 90%, and a single analysis, 488 subjects (143 in the untrained group, 202 in the compressions-only group, and 143 in the compressions and ventilation group) were required to detect 21% competency in the compressions-only group vs. 7% competency in the untrained group or 34% competency in the compressions and ventilations group.

### Statistical analyses

The primary analysis compared CPR competency of the compressions-only group with both the untrained group as well as the compressions and ventilations group using a Chi-Squared test for proportions with Bonferroni adjustment using a nominal p-value of 0.025 for significance to account for the double comparison. Secondary analyses involved similar double comparisons using Wilcoxon rank sum test when comparing medians, and the Chi-Squared test when comparing proportions, with Bonferroni adjustment. Sensitivity analysis evaluated alternative, less stringent cut points for CPR competency of 80%, 70%, 60% or 50% or greater ‘correct’ compressions. A post-hoc subgroup analysis compared CPR competency in those subjects who reported no prior training in CPR. All analyses were performed using S-Plus 6.0 software (Tibco Software Inc., Palo Alto, CA).

## Results

**Figure 1:**
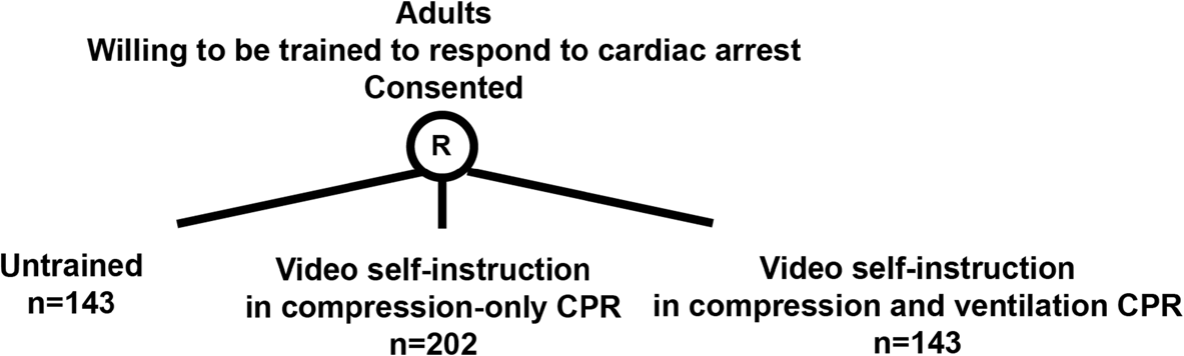
Study design and enrollment.

488 subjects were randomized. Of these, 143 were allocated to the untrained group, 202 to the compression-only group, and 143 to the compression and ventilation group (Figure 1). 49 subjects had CPR process data that was not retrievable from the mannikin. Of 439 subjects with analyzable data, 135 were allocated to the untrained group, 185 to the compressions-only group, and 119 to the compressions and ventilations group. Baseline characteristics of participating subjects are presented in Table 1.

**Table 1:** Baseline characteristics

**Characteristic**	**Overall (N = 439)**	**Untrained (N = 135)**	**Continuous compressions (N = 185)**	**Compressions and ventilations (N = 119)**
Age, mean (SD)	33.3 (17.0)	31.3 (16.0)	34.9 (17.3)	33.4 (17.5)
Male Gender, %	46.0	43.7	49.7	42.9
Race, %				
White	65.4	68.9	60.6	68.9
Black / African-American	2.3	3.0	2.7	0.8
Asian	24.4	17.0	30.3	23.5
American Indian / Alaska Native	2.3	3.0	2.2	1.7
Native Hawaiian / Pacific Islander	3.0	4.4	1.1	4.2
Other	1.8	3.0	1.6	0.8
Hispanic, %	3.6	4.4	0.6	7.6
Education, %				
Eight grade or lower	18.5	21.5	15.1	20.2
Some high-school, no diploma	18.2	21.5	16.8	16.8
High-school diploma	11.2	9.6	11.4	12.6
Some college	27.3	23.0	29.2	29.4
Bachelor’s Degree	17.1	17.8	19.0	13.5
Graduate degree	6.4	5.9	5.9	7.6
CPR training, ever, %	62.2	61.5	63.2	61.3
Performed CPR, ever, %	8.0	6.7	9.7	6.7
History of heart disease, self, %	3.6	2.2	4.3	4.2
History of heart disease, 1^st^ degree family, %	29.8	32.6	23.2	37.0
DVD player at home, %	95.2	97.8	91.9	97.5

25 (5.7%) achieved ‘competency in CPR’, i.e. at least 90% of compressions had correct rate, depth and hand position. Four (3%) achieved competency in the untrained group, nine (4.9%) in the compressions-only group, and 12 (10.1%) in the compressions and ventilations group. There was no significant difference in competency between the compressions-only group and the untrained group (χ^2^ = 0.32; df = 1; p-value = 0.57), or between the compressions-only group and the compressions and ventilations group (χ^2^ = 2.31; df = 1; p-value = 0.13) (Table 2).

**Table 2:** Competency in CPR by training group

	**Overall (N = 439)**	**Untrained (N = 135)**		**Continuous compressions (N = 185)**	**Compressions and ventilations (N = 119)**	
	**n (%)**	**n (%)**	**Comparison to continuous compressions**	**n (%)**	**n (%)**	**Comparison to continuous compressions**
Achieving **90%** Correct Compressions	25 (5.7)	4 (3.0)	Chi-Square = 0.32 df = 1 p-value = 0.57	9 (4.9)	12 (10.1)	Chi-Square = 2.31 df = 1 p-value = 0.13
Achieving **80%** Correct Compressions	30 (6.8)	7 (5.2)	Chi-Square = 2.07 df = 1 p-value = 0.15	19 (10.3)	14 (11.8)	Chi-Square = 0.05 df = 1 p-value = 0.83
Achieving **70%** Correct Compressions	66 (15.0)	13 (9.6)	Chi-Square = 2.77 df = 1 p-value = 0.10	31 (16.7)	22 (18.5)	Chi-Square = 0.05 df = 1 p-value = 0.82
Achieving **60%** Correct Compressions	98 (22.3)	23 (17.0)	Chi-Square = 1.22 df = 1 p-value = 0.27	42 (22.7)	33 (27.7)	Chi-Square = 0.73 df = 1 p-value = 0.39
Achieving **50%** Correct Compressions	129 (29.4)	33 (24.4)	Chi-Square = 0.32 df = 1 p-value = 0.57	55 (29.7)	41 (34.5)	Chi-Square = 2.31 df = 1 p-value = 0.13

A greater proportion of subjects demonstrated less stringent degrees of competency in each group, although there were no significant differences in competency between groups (Table 3). Overall, subjects achieved a median of 20.7% correct compressions (IQR: 0.3%, 57.8%), a median of 11.3% (IQR: 0%, 51.3%) in the untrained group, 23.6% (IQR: 1%, 57.9%) in the compressions-only group, and 30.8% (IQR: 3%, 61.6%) in the compressions and ventilations group. There was no significant difference in CPR competency of the compressions-only group compared to the compression and ventilations group (Z = −0.9; p-val = 0.36), but the compressions-only group tended to achieve greater competency than the untrained group (Z = −2.2; p-value = 0.027) (Table 3).

**Table 3:** Components of compressions by training group

	**Overall (N = 439)**	**Untrained (N = 135)**		**Continuous compressions (N = 185)**	**Compressions and ventilations (N = 119)**	
	**Median (IQR)**	**Median (IQR)**	**Comparison to continuous compressions**	**Median (IQR)**	**Median (IQR)**	**Comparison to continuous compressions**
Proportion Correct Compressions	20.7 (0.3, 57.8)	11.3 (0.0, 51.3)	Z = −2.20 p-value = 0.028	23.6 (1.0, 57.9)	30.8 (3.0, 61.6)	Z = −0.91 p-value = 0.36
Average Compression Rate, no./min.	94.0 (72.0, 108.0)	85.0 (62.0, 102.8)	Z = −1.32 p-value = 0.187	93.0 (65.0, 105.5)	104.0 (90.2, 113.0)	Z = −5.14 p-value < 0.001
Compression Depth in mm	37.0 (26.0, 44.0)	36.0 (20.0, 44.0)	Z = −0.48 p-value = 0.63	37.0 (27.0, 43.0)	38.5 (29.2, 45.0)	Z = −1.70 p-value = 0.09
Proportion Compression Depth > 38 mm	51.3 (4.2, 93.6)	55.0 (0.6, 93.1)	Z = −0.60 p-value = 0.55	55.7 (5.9, 89.1)	63.2 (12.9, 96.9)	Z = −1.10 p-value = 0.27
Proportion with Correct Hand Placement	89.2 (58.8, 99.7)	77.3 (49.0, 97.2)	Z = −3.52 p-value = <0.001	93.5 (62.3, 100.0)	91.8 (71.4, 99.7)	Z = 0.19 p-value = 0.85

**Figure 2:**
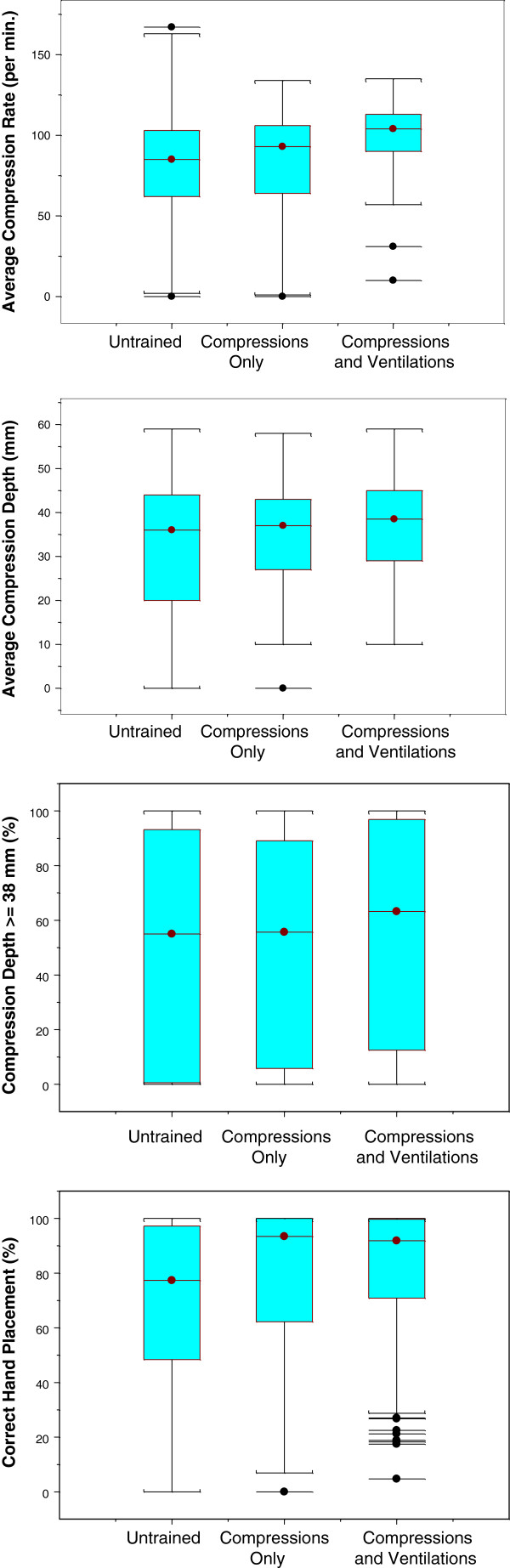
Box Plots of Individual Compression Components.

Each component of CPR was considered separately (Table 3 and Figure 2). The median of individual average compression rates was 94 (IQR: 72, 108) overall, 84 (IQR: 62, 103) for the untrained group, 93 (IQR: 65, 105) for the compressions-only group, and 104 (IQR: 90, 113) for the compressions and ventilations group. While the compression rate did not differ significantly between the untrained group and the compressions-only group (Z = −1.3; p-value = 0.2), the compressions and ventilations group achieved significantly higher compression rate than the compressions-only group (Z = −5.1; p-value < 0.0001).

Individual average compression depth (in mm) was median 37 (IQR: 26, 44) overall, 36 (IQR: 20, 44) for the untrained group, 37 (IQR: 27, 43) for the compressions-only group, and 38.5 (IQR: 29, 45) for the compressions and ventilations group. The compressions-only group did not have a significantly different compression depth compared to either the untrained group (Z = −0.5; p-value = 0.63) or the compressions and ventilation group (Z = −1.7; p-value = 0.09). Each group achieved a similar proportion of compressions with depth ≥ 38 mm (Table 3).

Correct hand placement was the compression component that was best performed overall, with a median proportion of 89.2% (IQR: 58.8%, 99.7%). The proportion of compressions with correct hand placement was median 77.3% (IQR: 49%, 97.2%) in the untrained group, 93.5% (IQR: 62.3%, 100%) in the compressions-only group and 91.8% (IQR: 71.4%, 99.7%) in the compressions and ventilations group. In this category, performance in the compressions-only group was similar to that in the compression and ventilations group (Z = 0.19; p-value = 0.85), but significantly better than in the untrained group (Z = −3.5; p-value = 0.0004).

Among subjects who reported that they had never been trained in CPR, there were no significant differences in CPR competency between the compressions-only group and the untrained group or the compressions and ventilations group.

## Discussion

We observed a low rate of competency in CPR skills among lay persons not trained in CPR in the last year, with or without video-self instruction using a personal manikin. There was no significant difference in competency, defined as at least 90% of compressions with correct rate, depth and hand position. The compressions-only group demonstrated greater competence for some components of CPR than the untrained group, particularly the proportion of correct compressions and the proportion of compressions with correct hand position.

Video self-instruction in CPR was developed to reduce the time necessary to learn these potentially life-saving skills. Traditional CPR courses are posited to provide an excess of information that is not vital to the basic main steps of CPR. These extra steps are posited to confuse the learners, ultimately reducing the competency of CPR knowledge [11]. Laypersons are able to use video self-instruction to voluntarily learn CPR in the comfort of their own home. This benefits those members of the public who might withdraw from practicing new skills in a public area. VSI in CPR allows learners to work at their desired pace, so that they can go back and review concepts without feeling like they are holding anyone up. Furthermore, with a condensed 10-minute video, laypersons may be more willing to complete the training without having to devote a large amount of their day. Overall, these benefits increase the potential for the general public to learn lifesaving CPR skills. With a well-trained general public able to perform accurate CPR, the chance of survival for cardiac arrest could ultimately increase. It appears that survival after out-of-hospital cardiac arrest is indeed improving over time in the United States [1,12] coincident with broad promotion of use of compressions-only CPR by lay persons. (http://www.handsonlycpr.org accessed on May 25, 2011) It remains unclear whether this temporal association is also a causal association. Given the magnitude of the burden of cardiac arrest, ongoing efforts are warranted to try to continue to improve survival as well as to understand reasons for the improvement in outcome.

Current guidelines for CPR recommend a compression depth of at least two inches, which was more than was evaluated in this study. The present study suggests that it may be difficult for lay persons with minimal training to achieve such a compression depth. Nearly 80% of the population in the study community report that they have received training in CPR skills at some point [13]. However in most communities, a minority of the population have been trained in CPR [14]. Therefore we believe that the most appropriate comparison for simplified training in CPR skills is no training at all, since that is what individuals have received to date in most communities.

Prior randomized trials have evaluated educational interventions that use a variety of media and intervention durations for self-instruction of lay persons in CPR skills [8,15-22]. Each of these enrolled fewer subjects than the present study. Collectively the prior studies and the present one suggest that video self-instruction can achieve small improvements in lay persons CPR skills compared to no training, which may or may not be sufficient to improve survival if such skills are later used to provide bystander CPR to persons in cardiac arrest. But neither the prior studies nor the present one assessed whether training lay persons in CPR is associated with salutary benefits including earlier recognition and response to cardiac arrest that would also improve survival.

Promulgation of compressions-only CPR by laypersons is intended to improve coronary blood flow by increasing provision of any compressions as well as by decreasing interruptions of compressions when they are performed. Coronary perfusion pressure (CPP), which is the difference between the aortic diastolic pressure and the right atrial pressure, is correlated with restoration of spontaneous circulation in animals [23] and humans [24]. Adequate CPP takes time to develop once chest compressions are initiated, and quickly dissipates in the absence of effective and continuous external chest compressions [25]. Interruptions in chest compressions, such as to provide ventilations, decrease CPP and consequent likelihood of a successful outcome [5]. The magnitude of CPP achieved during resuscitation is correlated with the quantity and quality of external chest compressions [26]. CPR training is intended to enhance delivery of those components of CPR that improve CPP and survival.

Potential disadvantages of compressions-only CPR include the withholding of ventilation from patients with cardiac arrest of non-cardiac (e.g. respiratory) etiology, and consequent reduced survival in this subgroup [27-29].

There were some limitations to how CPR skills were assessed in this trial. Competency was assessed by sensored manikins rather than by direct observation by instructors in this study. Greater chest compression fraction, defined as the proportion of time in which chest compressions are performed, is correlated with greater survival to discharge among patients who present with a shockable rhythm [30], and greater restoration of spontaneous circulation in patients with other rhythms [31]. Greater depth of compression [32], briefer perishock pauses [33], and optimal compression rates are associated with better outcomes in patients with out-of-hospital cardiac arrest [34]. Although it is reassuring that the compressions-only group had a greater proportion of correct chest compressions, the mannikins that were used did not evaluate compression fraction, or compressions in relation to use of an automated external defibrillator, or the presence or absence of complete chest recoil, or the adequacy of ventilations when they were performed. As well, skills were tested immediately rather than a month or longer after training, at which time some skill decay may have occurred. Thus it is unclear whether the skills obtained by the brief training evaluated in the present study would result in improved restoration of circulation or survival.

Other weaknesses of this study were the use of a restrictive primary endpoint for CPR quality (simultaneous combination of adequate depth, rate and hand placement). Skill competency was assessed for five minutes, which may have been associated with greater fatigue and skill degradation compared to other studies that used a two-minute assessment period [17]. Importantly subjects were evaluated immediately after brief rather than sustained watch and practice, which may have reduced participants’ competency. Each of these factors may have contributed to the low rate of competency achieved overall in each group. Greater skills were observed when we considered less stringent definitions of competency, although there were still no differences between groups. We recruited among lay persons in the community rather than among those with self-interest in CPR because of personal exposure to heart disease [18], so participants may have had less motivation to achieve skill competence in the present study.

This study has some strengths. Its large sample size provided sufficient power to detect smaller differences in CPR process than prior studies. The randomized nature of the study design, standardized nature of exposure to the intervention, and objective measurement of components of CPR using sensored mannikins were intended to reduce bias. Finally, alternative cut points for assessment of skill competence were considered in place of the restrictive primary endpoint for CPR quality.

## Conclusions

VSI in compressions-only CPR did not achieve greater overall competency but did achieve some CPR skills better than without training. Ongoing research is necessary to understand the relationship between lay persons’ CPR skills, their performance of bystander CPR in the field, and patient survival after cardiac arrest.

## Abbreviations

CPR: Cardiopulmonary resuscitation; VSI: Video-self instruction; OHCA: Out-of-hospital cardiac arrest; IQR: Interquartile range; CPP: Coronary perfusion pressure.

## Competing interests

The authors declare that they have no competing interests.

## Authors’ contributions

RG acquired the data, or analysis and participated in drafting the manuscript; EH participated in the design and analysis of the data, and critical revisions of the manuscript. DF contributed to interpretation of the data and critical revisions of the manuscript; GN conceived of the study, and participated in its design and coordination and drafted the manuscript. All have given final approval of the version to be published. The funding bodies had no role in the design, collection, analysis or interpretation of the data nor in the writing of the manuscript or in the decision to submit the manuscript for publication. All authors read and approved the final manuscript.
